# High-contiguity genome assembly of moth bean (*Vigna aconitifolia*) reveals genes for disease resistance and flavonoid biosynthesis

**DOI:** 10.3389/fpls.2026.1932838

**Published:** 2026-09-21

**Authors:** Adhimoolam Karthikeyan, Gunasekaran Ariharasutharsan, Sankaran Divya, Kantharaj Vimalraj, Natesan Senthil, Thamilarasan Senthil Kumar, Manoharan Akilan

**Affiliations:** 1 Department of Biotechnology, Centre of Innovation, Agricultural College and Research Institute, Tamil Nadu Agricultural University, Madurai, India; 2 Subtropical Horticulture Research Institute, Jeju National University, Jeju, Republic of Korea; 3 Department of Plant Molecular Biology and Bioinformatics, Centre for Plant Molecular Biology and Biotechnology, Tamil Nadu Agricultural University, Coimbatore, India; 4 School of Biological and Medical Science, Oxford Brookes University, Oxford, United Kingdom; 5 Institute of Agriculture and Life Science (IALS), Gyeongsang National University, Jinju, Republic of Korea; 6 Genomics Division, National Institute of Agricultural Sciences, Rural Development Administration, Jeonju, Republic of Korea; 7 Department of Genetics and Plant Breeding, Anbil Dharmalingam Agricultural College and Research Institute, Tamil Nadu Agricultural University, Trichy, India

**Keywords:** flavonoids, high quality genome, moth bean, R genes, *Vigna* spp.

## Abstract

Moth bean (*Vigna aconitifolia*), a protein-rich, arid-adapted legume of considerable agronomic importance, lacks a whole genome assembly, limiting genome-enabled trait discovery and genetic improvement. In this study, we estimated its genome size using 31.28 Gb of Illumina short-read sequencing data, and a k-mer analysis (k = 21) predicted a genome size of 448.98 Mb. We generated a highly contiguous 420.3 Mb genome assembly using 37.04 Gb of PacBio HiFi long-read sequencing data, corresponding to 82.5× genome coverage. The assembly achieved a contig N50 of 22.30 Mb, with a maximum contig length of 59.6 Mb, and exhibited >98% completeness based on BUSCO assessment. Comparative genomic and phylogenetic analyses of 14 closely related species identified *V. radiata* and *V. mungo* as the closest extant relatives of moth bean, with an estimated divergence time of ~3.7 Mya, consistent with established phylogenetic relationships within the genus *Vigna*. Structural annotation predicted ~27,434 protein-coding genes, of which 27,411 (99.91%) were functionally annotated. Repetitive sequences accounted for 50.66% of the genome, with retrotransposons representing the predominant class and occupying 109.9 Mb (26.1%). We further identified 178 disease resistance–related genes and 54 genes involved in flavonoid biosynthesis, including those encoding key enzymes such as chalcone synthase, chalcone isomerase, flavanone 3-hydroxylase, and flavone synthase II. Collectively, we present the first highly contiguous moth bean genome assembly, providing a foundational resource for evolutionary, functional genomics and breeding studies. This resource facilitates characterization of genes governing disease resistance and flavonoid biosynthesis, supporting genetic improvement of stress tolerance and nutritional quality in moth bean.

## 1 Introduction

Moth bean [*Vigna aconitifolia* (Jacq.) Marechal] is one of the least studied and underutilized *Vigna* species despite its considerable agronomic potential. Although its cultivation remains limited because of agronomic, cultural, and economic constraints, moth bean has attracted increasing attention owing to its nutritional value, nitrogen-fixing ability, adaptation to marginal soils, and resilience to abiotic stress. Its seeds are rich in protein, dietary fiber, minerals, vitamins, carbohydrates, and polyphenols, making them an important source of human food and animal feed (Kanishka et al., 2023; Dhull et al., 2024). Despite these valuable properties, the crop remains underutilized relative to other *Vigna* species and is cultivated primarily within traditional farming systems (Saxena et al., 2023; Singh et al., 2024). Moth bean is a self-pollinated diploid species (2n = 2x = 22) with an estimated genome size of approximately 469 Mbp (Diakostefani et al., 2024). Both wild and cultivated forms originated on the Indian subcontinent, which represents the primary center of diversity for this orphan legume. Germplasm resources are conserved in international gene banks and by smallholder farmers, with the largest collection maintained at the National gene bank, ICAR-NBPGR, India (Bisht and Singh, 2013; Kanishka et al., 2023). A recent review by Kanishka et al. (2023) highlighted that research on germplasm conservation and characterization, whereas breeding efforts have largely targeted improvements in yield, stress resilience and nutritional quality. Nevertheless, genetic improvement has remained modest, and breeding progress has been relatively slow.

Moth bean is highly susceptible to economically important diseases and insect pests, including yellow mosaic disease, powdery mildew, and bruchids, which substantially compromise seed yield and quality. Developing resistant cultivars is therefore a major breeding objective. However, the genetic architecture and regulatory mechanisms underlying disease resistance, particularly the diversity and evolution of resistance (*R)* genes, remain poorly understood. Whole-genome sequencing analysis has become an effective approach for identifying candidate R genes, elucidating their evolutionary history, and accelerating the development of disease-resistant cultivars (Jegadeesan et al., 2021; Savadi et al., 2022). Moreover, flavonoids are an important plant secondary metabolites that contribute to pigmentation, defense responses, and tolerance to environmental stresses. In moth bean, flavonoid biosynthesis is likely to play a critical role in environmental adaptation while enhancing the nutritional and antioxidant properties of this stress-resilient legume. However, the genetic basis of flavonoid biosynthesis remains poorly understood, highlighting the need to identify and characterize the genes involved in this pathway.

Advances in genome sequencing, assembly and annotation approaches have transformed plant genomics research, providing unprecedented opportunities for genomics-assisted breeding and improving our understanding of the genetic architecture underlying agronomically important traits, including disease resistance, abiotic stress tolerance, and nutritional quality (Yuan et al., 2025; Zhuang et al., 2026). Consequently, genomics resources for several *Vigna* species have expanded considerably, including genome assembly for 500.14-Mb mung bean (*V. radiata*) with a contig N50 of 46 Mb (Jia et al., 2025), 499-Mb black gram (*V.mungo*) with a contig N50 of 5.2 Mb (Pootakham et al., 2021), 519.43-Mb cowpea (*V. unguiculata*) with a contig N50 of 10.91 Mb (Lonardi et al., 2019), 465.19-Mb rice bean (*V. umbellata*) with a contig N50 of 18.26 Mb (Guan et al., 2022) and 447.8-Mb adzuki bean (*V. angularis*) with a contig N50 of 16.5 Mb (Li et al., 2024). However, genomic resources for moth bean remain limited, and the absence of whole genome sequence and comprehensive genome annotation has constrained molecular breeding, comparative genomics, and functional genomics studies. Here, we present the highly contiguous genome assembly and a comprehensive genome annotation of the moth bean for the first time. We further characterized disease resistance (R) genes, and genes involved in flavonoid biosynthesis and investigated the evolutionary relationships of moth bean with other *Vigna* species and representative legumes. These genomic resources provide a valuable foundation for genome evolution, functional studies, and genomic-assisted breeding aimed at accelerating the genetic improvement of this important climate-resilient crop.

## 2 Materials and methods

### 2.1 Plant genetic materials and nucleic acids isolation

The moth bean cultivar TMV (Mb) 1, a widely cultivated variety in India developed through pure-line selection from the Villupuram local landrace of Tamil Nadu, India was used in this study. Seeds were sourced from the Oil seeds Research Station, Tamil Nadu Agricultural University, Tindivanam, India. Moth bean plants were grown under the green house at the Agriculture college and research institute, Tamil Nadu Agricultural University, Madurai, India. High-molecular-weight genomic DNA was isolated from young leaves using the DNeasy Plant Mini kit (Qiagen) and used for both long and short read DNA sequencing. Total RNA was isolated from roots, leaves, flowers, pods, and seeds using the RNeasy Plant Mini Kit. Residual DNA was removed by treatment with RNasefree DNase (Qiagen). RNA concentration (ng/μL) and purity (A260/280) were assessed using a bio spectrometer (Eppendorf, Hamburg, Germany). RNA integrity (> 6) was confirmed using an Agilent Bioanalyzer 2100 system (Agilent Technologies, Santa Clara, CA, USA). Only high-quality RNA was used for RNA sequencing (RNA-seq) and quantitative real time PCR (qRT–PCR) analyses.

### 2.2 Library preparation and sequencing analysis

A high-fidelity (HiFi) library for long-read DNA sequencing was prepared using the SMRTbell Express Template Prep Kit 2.0 (Pacific Biosciences), targeting an average insert size of 18–20 kb. Sequencing was performed in circular consensus sequencing (CCS) mode on the PacBio Revio system. HiFi reads were generated from the subreads using the CCS algorithm in SMRT Link v13.1 (PacBio), retaining reads with a minimum of 3 full passes (min passes = 3) and a predicted consensus accuracy ≥ 99% (min RQ = 0.99, i.e. read quality ≥ Q20). The resulting HiFi reads were subsequently used for contig-level genome assembly. For short-read DNA sequencing, an illumina library with an average insert size of 350 bp in length was constructed and sequenced on the Illumina NovaSeq 6000 platform. For RNA sequencing, libraries with an average insert size of 150 bp were prepared using the TruSeq Stranded mRNA Library Prep Kit (Illumina) and sequenced on the Illumina NovaSeq 6000 platform. All Illumina-generated raw reads were quality filtered using Fastp (v1.0.1), and the resulting high-quality reads were retained for downstream analyses.

### 2.3 Genome size estimation, assembly and quality assessment

Genome size was estimated from Illumina short DNA reads using a *k*-mer-based approach. Briefly, 21-mer frequencies were counted using Jellyfish v2.3.1 (https://github.com/gmarcais/Jellyfish) (Marçais and Kingsford, 2011), and the *k*-mer frequency distribution was analysed with GenomeScope 2.0 v2.0 (http://genomescope.org/genomescope2.0/) (Ranallo-Benavidez et al., 2020) to estimate genome size, heterozygosity, and repeat content. Primary contigs were assembled from PacBio HiFi reads using hifiasm v0.25.0 with default parameters (Cheng et al., 2021). Redundant haplotypic sequences were subsequently removed using Purge_dups (v1.2.6) (https://github.com/dfguan/purge_dups). The final assembly was designated Vac-TMV1.v1.0 to indicate the species, accession, and assembly version. Assembly statistics, including contig number, contig N50, and GC content, were generated using QUAST (v 5.3.0) (Gurevich et al., 2013). Genome assembly quality and completeness were evaluated using three complementary approaches. First, illumina short-DNA reads were aligned to the assembled genome using BWA v0.7.19 (Li and Durbin, 2009), and sequencing depth across the assembly was calculated using SAMtools v1.22.1. Second, RNA-seq reads generated from different tissues were aligned to the genome using HISAT2 v2.2.1 (Kim et al., 2015). Third, genome completeness was evaluated using BUSCO v5.3.2 (Simao et al., 2015) with the *embryophyta_odb10*, *eudicots_odb10*, and *fabales_odb10* datasets.

### 2.4 Genome annotation

Repetitive elements in the moth bean genome were identified using a combined *de novo* and homology-based approach. A *de novo* repeat library was constructed using RepeatModeler (v2.0.7) (https://www.repeatmasker.org/dev/RepeatModeler/) with the LTRstruct pipeline (Flynn et al., 2020) enabled. This pipeline integrates RECON (v1.08), RepeatScout (v1.0.5), LTR harvest, and LTR_retriever to identify repetitive elements, delineate their boundaries, and generate consensus sequences representing interspersed repeats. For homology-based repeat annotation, RepeatMasker v4.2.1 (Smit et al., 2013-2015) was used to identify transposable elements (TEs) by aligning the genome assembly against the plant repeat sequences available in RepBase. The *de novo* repeat library generated by RepeatModeler and the RepBase repeat library were combined to create a comprehensive repeat library, which was subsequently used by RepeatMasker to annotate repetitive elements and soft-mask the genome. To evaluate the continuity of long terminal repeat (LTR) retrotransposons and the quality of repeat assembly, the LTR Assembly Index (LAI) was calculated using LTR_retriever.

Gene models were predicted from the repeat-masked genome using a combined strategy integrating *ab initio*, homology-based, and RNAseq-guided approaches via the BRAKER3 pipeline (v3.0.8) (Gabriel et al., 2024). Gene prediction was initiated with GeneMark-ETP (Bruna et al., 2024), which incorporates RNA-seq data and protein homology evidence to train prediction models and generate preliminary gene structures. A high-confidence subset of these gene models was subsequently used to train the *ab initio* gene predictor AUGUSTUS (Stanke et al., 2006). The final consensus gene models were obtained by integrating the predictions from GeneMark-ETP and AUGUSTUS using TSEBRA (Gabriel et al., 2021), which selects the most reliable transcript models based on supporting evidence. RNA-seq data from different tissues of the cv. TMV (Mb) 1 were used to provide RNAseq-evidence for gene prediction. Protein homology evidence was obtained from closely related *Vigna* species were combined with the Viridiplantae protein dataset from OrthoDB and provided to BRAKER3 via the --prot_seq parameter. To improve annotation quality and completeness, the --busco_lineage = fabales_odb10 option was enabled within BRAKER3 for BUSCO-based gene model assessment. Additionally, GUSHR (v1.0.0; https://github.com/Gaius-Augustus/GUSHR) was used in conjunction with RNA-seq data to annotate untranslated regions (UTRs), further refining gene structure definitions. Functional annotation of protein-coding genes was performed using BLASTP and BLASTN searches against multiple public databases with an E-value threshold of ≤ 1 × 10^-5^. The queried databases included the NCBI non-redundant protein (NR) and nucleotide (NT) databases, UniProtKB, EggNOG, InterPro, Gene Ontology (GO), and KEGG databases. Similarity searches were conducted using the same E-value threshold to identify statistically significant homologs. Transcription factors (TFs) were identified using PlantTFDB 4.0 (Jin et al., 2017). Non-coding RNA (ncRNAs) were annotated using specialized prediction tools. tRNAs were identified using tRNAscan-SE v2.0.12 (Chan et al., 2021) with the ‘eukaryote’ model, whereas rRNAs were detected using Barrnap v0.9 (https://github.com/tseemann/barrnap). miRNAs and snRNAs were identified using INFERNAL v1.1.5 (Nawrocki and Eddy, 2013) with covariance models from Rfam database (release 14.5). Pseudogenes were predicted using GenBlastA v1.0.4, followed by structural refinement with GeneWise v2.4.1.

### 2.5 Comparative genomics analyses

Orthologous gene families were identified using *OrthoFinder* v2.4.0 (Emms and Kelly, 2019) based on the protein sequences of moth bean TMV (Mb)1 and 14 additional species, including *V. radiata*, *V. mungo*, *V. unguiculata*, *V. angularis*, *V. umbellata*, *V. stipulacea*, *V. trilobata*, *Vicia faba*, *Cajanus cajan*, *Lotus japonicus*, *Pisum sativum*, *Cicer arietinum*, *Arabidopsis thaliana*, and *Oryza sativa*. Single-copy orthologs were aligned using MUSCLE v5.1 (Edgar, 2004), and poorly aligned regions were removed using trimAl v1.4 rev15 with the -automated1 option. The resulting alignments were concatenated using catsequences (https://github.com/ChrisCreevey/catsequences). The best-fitting substitution model (JTT+I+G4+F) was selected using ModelTest-NG v0.1.7 (Darriba et al., 2020). A maximum-likelihood phylogenetic tree was constructed using RAxML-NG v1.0.2 (Kozlov et al., 2019) with 100 bootstrap replicates, using *O. sativa* as the outgroup. Species divergence times were estimated using the MCMCtree program implemented in PAML v4.10.10 (Yang, 2007) under an independent-rates relaxed molecular clock model with the approximate likelihood method. Time calibration was based on a fossil-based constraint at the papilionoid legume crown node (56–60 Mya) (Lavin et al., 2005) and a broad constraint at the root. Gene family expansion and contraction were analysed using CAFE v5 (Mendes et al., 2020) based on maximum-likelihood estimation of the birth–death parameter (λ), with an estimated error model; families with p < 0.05 were considered significantly changed. Functional enrichment analyses of expanded and contracted gene families were performed using GO and KEGG annotations obtained from eggNOG-mapper.

### 2.6 Gene identification and transcriptome analysis

Genes containing the TIR (PF01582), RPW8 (PF05659), and NB-ARC (Pfam: PF00931) domains were classified as nucleotide-binding leucine-rich repeat (NLR) genes according to established domain based criteria. The longest coding sequence (CDS) isoform of each predicted gene was used for NLR identification by integrating the results of NLR-Annotator v2.1b and Pfam annotations generated using InterProScan. The presence of NLR-associated domains was further validated, and NLR subfamilies were classified using the NCBI-CDD. To investigate tissue-specific expression, RNA-seq reads from root, leaf, flower, pod, and seed tissues were aligned to the moth bean reference genome using HISAT2. Gene-level read counts were generated using feature Counts, and transcript abundance was normalized as transcripts per million (TPM) using TBtools. Multiple sequence alignment of NLR protein sequences was performed using the E-INS-i algorithm implemented in MAFFT v7.520. A neighbor-joining phylogenetic tree was constructed using TreeBeST v1.9.2 with1000 bootstrap replicates (nj -b 1000 -W). The phylogenetic tree was visualized together with TPM expression value (Log2 (TPM + 1)) using the ggtree package in R. Reference protein sequences of enzymes involved in flavonoid biosynthesis were obtained from *Arabidopsis thaliana* and closely related legume species, including *Glycine max*, *V. radiata*, *V.angularis*, *V.unguiculata*, and *V.umbellata*, through the KEGG database. Protein sequences were retrieved using the KEGG API (https://www.kegg.jp/kegg/rest/keggapi.html) based on flavonoid biosynthesis pathway annotations and related metabolic pathways. The retrieved protein sequences were used as queries for BLASTP searches (E-value ≤ 1e−5) against the moth bean predicted protein dataset with an E-value threshold of ≤ 1 × 10^-5^ to identify putative homologs involved in flavonoid biosynthesis. To investigate tissue-specific expression patterns, RNA-seq data from root, leaf, flower, pod, and seed tissues were analysed. Gene expression levels were quantified as transcripts per million (TPM), and log_2_ (TPM +1) values were used for downstream visualization. Normalized expression values were showed as heat maps to compare tissue-specific expression profiles of flavonoid biosynthesis genes. These expression patterns subsequently were integrated into the flavonoid biosynthesis pathway to illustrate the relative expression levels of the corresponding enzymes.

### 2.7 qRT-PCR analysis

Total RNA was converted to first-strand cDNA using the iScript™ cDNA Synthesis Kit (Bio-Rad Laboratories, Hercules, CA, USA) following user guidelines. qRT–PCR was conducted on a Bio-Rad CFX96 system (Bio-Rad Laboratories). Gene specific primers were designed using Primer Premier 6.0 (**Supplementary Table 1**). Each 20 µL reaction contained 2.5 µL of first-strand cDNA, 1.0 µL of gene-specific primers (10 µM), 10 µL of AccuPower^®^ 2× GreenStar™ SYBR Green Master Mix (Bio-Rad), and 6.5 µL of nuclease-free water. Each reaction was performed with three independent biological replicates. Actin was used as the internal reference gene (Suranjika et al., 2022), and relative gene expression levels were calculated using the 2^−ΔΔCT^ method.

## 3 Results

### 3.1 High-contiguity genome assembly

A total of 31.28 Gb of Illumina short reads were generated to estimate the genome size (**Supplementary Table 2**). K-mer analysis (k = 21) identified 1256517795 k-mers with a major peak at a depth of 74, estimating the genome size at 448.98 Mb with a heterozygosity rate of 0.13% (**Supplementary Table 3**; **Figure 1A**). For genome assembly, 2026331 PacBio HiFi reads (~37.04 Gb, ~82.5× coverage) were generated using the PacBio Revio platform, with maximum and average read lengths of 65.17 kb and 18.28 kb, respectively (**Supplementary Table 2**). *De novo* assembly with hifiasm resulted the final genome assembly (Vac-TMV1.v1.0), comprising 188 contigs spanning 420.36 Mb (93.63% of the estimated genome size), with a contig N50 of 22.30 Mb (**Table 1**). The longest contig was 59.65 Mb, and 90% of the assembled genome was contained within 21 contigs exceeding 5 Mb. Assembly quality was supported by a BUSCO completeness score exceeding 98% (**Table 2**). Furthermore, illumina DNA short reads (~31.28 Gb) and RNA-seq data (34.54 Gb) from different tissues mapped to the assembly at rates of ~99.63% and ~97.04%, respectively (**Supplementary Table 4****),** confirming its high completeness and accuracy.

**Figure 1:**
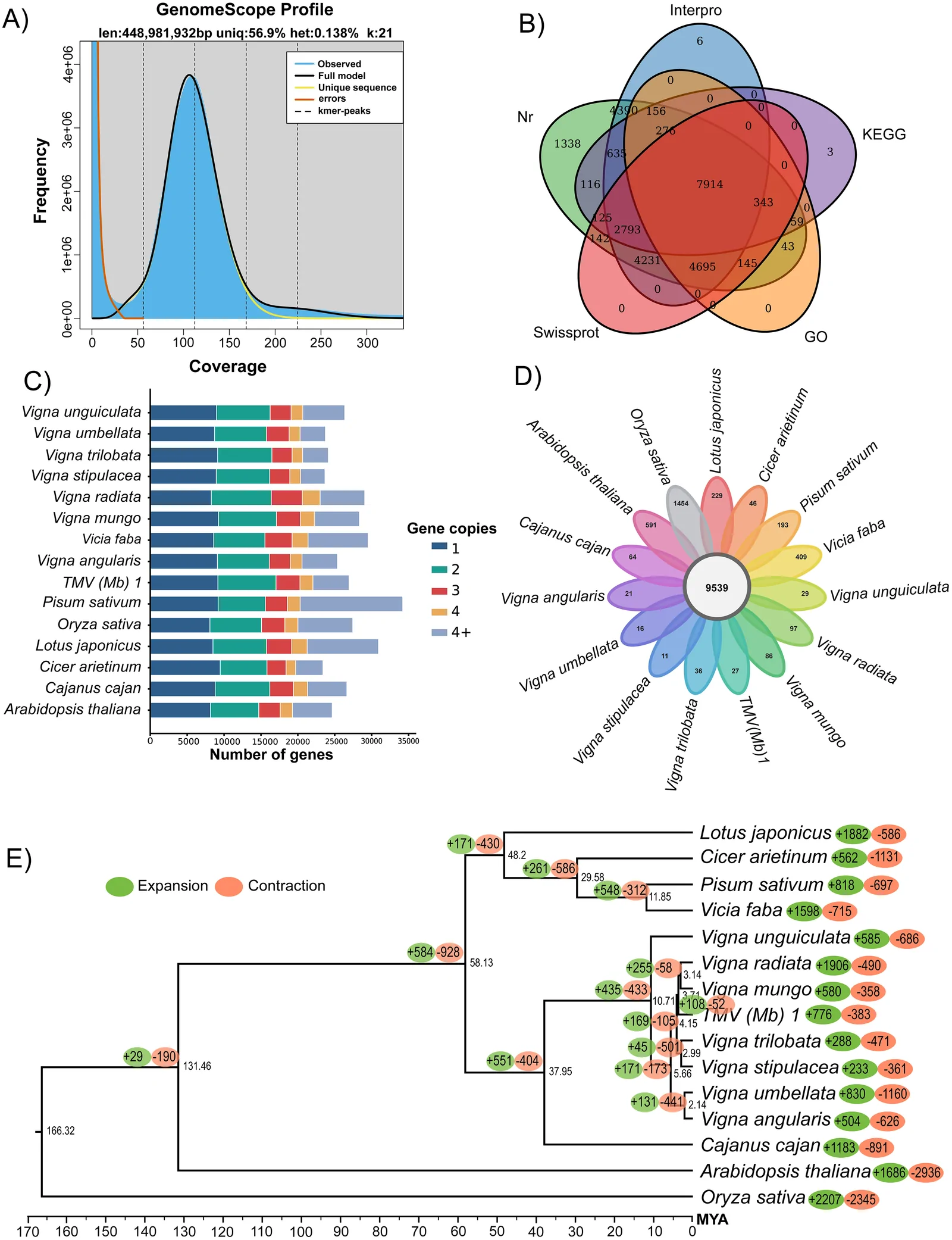
Genome characterization and comparative genomic analysis of *Vigna aconitifolia* cv TMV (Mb) 1. **(A)** 21-mer frequency distribution used for genome size estimation, **(B)** Venn diagram showing unique and shared gene annotations obtained from different databases, **(C)** Copy number distribution of gene families among the 15 related plant species, **(D)** Petal diagram illustrating gene family distribution across 15 related plant species, and **(E)** Phylogenetic relationships of moth bean and related plant species inferred from single-copy orthologous proteins. Numbers at each node indicate estimated divergence times in million years ago (MYA). Green and orange branch labels represent expanded and contracted gene families, respectively.

**Table 1:** Moth bean cv TMV (Mb) 1 genome assembly and annotation statistics.

Feature	Statistics	Value
Assembly	Total length of contig sequence (bp)	420363393
	Total number of contigs	188
	N50 of contigs (bp)	22307451
	N90 of contigs (bp)	5562970
	L50 of contig	7
	L90 of contig	21
	Minimum contig size (bp)	8158
	Maximum contig size (bp)	59653453
	GC content (%)	33.1
	AT	66.9
	N	0
Gene models	Number of protein coding genes	27434
	Number of transcripts	32680
	Average gene length (bp)	3314
	Average intron length (bp)	478
	Average CDS length (bp)	1259
	Average genes per contig	807
Gene annotation	Annotated genes	27411
	Percentage of annotated genes	99.91%

**Table 2:** BUSCO assessment of the moth bean cv TMV (Mb) 1 genome assembly and gene models.

Type	Embryophyta				Eudicots				Fabales			
	Genome		Gene		Genome		Gene		Genome		Gene	
	Number	Rate (%)	Number	Rate (%)	Number	Rate (%)	Number	Rate (%)	Number	Rate (%)	Number	Rate (%)
Complete BUSCOs (C)	1595	98.8	1605	99.4	2296	98.7	2301	98.9	5290	98.60	5332	99.4
Complete and single-copy BUSCOs (S)	1541	95.5	1539	95.4	2215	95.2	2223	95.6	5151	96.00	5172	96.4
Complete and duplicated BUSCOs (D)	54	3.3	66	4.1	81	3.5	78	3.4	139	2.6	160	3.0
Fragmented BUSCOs (F)	9	0.6	0	0.00	7	0.3	5	0.2	15	0.3	3	0.06
Missing BUSCOs (M)	10	0.6	9	0.6	23	1.0	20	0.9	61	1.1	31	0.60
Total BUSCO groups searched	1614		1614		2326		2326		5366		5366	

### 3.2 Genome annotation

A total of 212.9 Mb of repetitive sequences, representing 50.66% of the TMV1 genome, were identified (**Table 3**). Nine major classes of repetitive elements were annotated, with retrotransposons, comprising the largest fraction (109.9 Mb, 26.1%), including long terminal repeat (LTR) retrotransposons (108.02 Mb, 25.70%) and non-LTR retrotransposons (1.93 Mb, 0.46%). The LTR fraction was dominated by Gypsy (59.10 Mb, 14.06%) and Copia (46.02 Mb, 10.95%) elements, whereas DNA transposons accounted for 31.2 Mb (7.43%) of the genome. In addition, 13.39 Mb (3.19%) of repetitive sequences remained unclassified. Integration of *de novo* gene prediction, homology-based annotation, and RNA-seq data from five tissues identified 27,434 high- confidence protein-coding genes represented by 32,680 transcripts (**Table 1**). The mean gene length, exon number, and coding sequence length were 3314 bp, 5.2, and 1259 bp, respectively. Among the predicted transcripts, 32,657 (99.93%) contained complete coding sequences with both start codon and stop codons. BUSCO analysis indicated that the protein-coding gene set is 99% complete (**Table 2** and **Supplementary Figure 1**). Functional annotation assigned, 27,411 (99.91%) to at least one public database (**Figure 1B**), with the highest annotation rates in the Nr (99.86%) and Interpro (91.48%) databases, followed by GO (49.69%) and KEGG (44.69%). A total of 1999 transcription factor genes belonging to 58 families were identified (**Supplementary Table 5**). The largest families were bHLH (180), MYB (179), ERF (152), C2H2 (121), WOX (121), and WRKY (94). In addition, 7752 ncRNAs were annotated, comprising 6,394 rRNAs, 938 tRNAs, 294 snRNAs, and 126 miRNAs (**Table 4**).

**Table 3:** Classification and annotation of repetitive sequences in the moth bean cv TMV (Mb) 1 genome.

Type	Length (bp)	Rate (%)
**Class I:Retrotransposon**	109965895	26.16
**LTR-Retrotransposon**	108026140	25.70
LTR/Copia	46021540	10.95
LTR/Gypsy	59100860	14.06
LTR-other	2903740	0.69
**Non-LTR Retrotransposon**	1939991	0.46
SINE	41359	0.01
LINE	1898396	0.45
**Class II: DNA Transposon**	31221548	7.43
MULE-MuDR	6994163	1.66
hobo-Activator	6714000	1.60
Tourist/Harbinger	943594	0.22
Tc1-IS630-Pogo	166377	0.04
DNA-other	16403414	3.91
**Low Complexity**	2807243	0.67
**Tandem repeat**	9924314	2.36
**Unclassified**	13397309	3.19
**Total content**	212974620	50.66
**Genome length**	420363393	–

Bold letters indicate the major repeat classes.

**Table 4:** Summary of non-coding RNA predicted in the moth bean cv TMV (Mb) 1 genome assembly.

ncRNA type		Number	Average length (bp)	Total length (bp)	Percentage of genome sequence
miRNA		126	122.70	15461	0.004
tRNA		938	75.08	70430	0.017
rRNA	18s	763	1711.78	1306092	0.311
	28s	812	3345.57	2716606	0.646
	5.8s	701	154.00	107952	0.026
	5s	4118	110.79	456231	0.109
snRNA		294	100.10	29432	0.007

### 3.3 Evolutionary and comparative genomic analyses

Comparative genomic analyses of moth bean and 14 related plant species assigned 26,934 moth bean genes (98.2%) to 15,118 gene families, including 9,539 conserved families shared across all species (**Figures 1C, D**) and 27 lineage-specific families unique to moth bean (**Supplementary Tables 6, 7**). A time-calibrated phylogeny constructed from 1,593 single-copy orthologs recovered the established *Vigna* relationships and placed moth bean as sister to the *V. radiata*–*V. mungo* clade, with an estimated divergence time of ~3.7 Mya (95% HPD: 2.7–5.1 Mya) (**Figure 1E**). Gene family evolution analysis identified 776 expanded and 383 contracted families in the moth bean genome (**Supplementary Tables 8**, **9**). Gene ontology enrichment analysis revealed that expanded gene families were enriched for molecular functions related to ribosomal structure and RNA processing, including structural constituent of ribosome, structural molecule activity, RNA helicase activity and RNA binding; cellular components including cytosolic ribosomal subunits, ribosome and ribonucleoprotein complex; and biological processes including translation, peptide biosynthetic process, ribosome biogenesis and gene expression (**Supplementary Figure 2A**). On the other hand, contracted families were enriched for molecular functions including calmodulin binding, protein dimerization activity, nuclease activity and transmembrane transporter activity; the cellular components actin cytoskeleton, plasma membrane and cell periphery; and biological processes including programmed cell death, immune response, response to external stimulus and taxis (**Supplementary Figure 2B**). KEGG pathway enrichment analysis indicated that expanded gene families were mainly associated with ribosome, RNA polymerase, proteasome, translation factors and circadian rhythm, whereas contracted families were enriched in metabolic pathways including taurine and hypotaurine metabolism, glycosphingolipid biosynthesis, terpenoid-quinone and diterpenoid biosynthesis, galactose metabolism, ABC transporters and amino acid metabolism (**Supplementary Figure 3**). Collectively, these patterns indicate expansion of translation- and protein-synthesis-related gene families alongside contraction of families associated with defense responses and specialized metabolism in the moth bean genome.

### 3.4 Disease resistance *(R)*-genes and their interaction with miRNAs

A total of 178 candidate NLR genes were identified using NLR-Annotator and InterProScan (**Supplementary Table 10**). Based on conserved domain architectures, the NLRs were categorized into four canonical subfamilies: coiled-coil NB-ARC leucine-rich repeat (CNL), Toll/interleukin-1 receptor NB-ARC leucine-rich repeat (TNL), RPW8-NB-ARC leucine-rich repeat (RNL) and NB-and-LRR only proteins (NL). Phylogenetic analysis resolved the 178 NLR proteins into four distinct subfamilies (**Figure 2A****).** Expression profiles of the NLR genes across five tissues (root, leaf, flower, pod, and seed) are shown in **Figure 2B**. The NLR landscape was dominated by the CNL and TNL classes, each comprising 65 genes (36.52%) (**Figure 2C**). Gene ontology enrichment analysis associated 22 NLRs with the term “response to biotic stimulus” (GO: 0009607). These genes were enriched for the molecular functions nucleotide binding, nucleoside phosphate binding, the cellular components cell periphery and membrane, and the biological processes defense response to symbiont, symbiont-induced defense-related programmed cell death, signaling and plant-type hypersensitive response (**Supplementary Figure 4A**). KEGG pathway analysis assigned 36 NLR genes to the “plant–pathogen interaction” pathway (ko04626). These genes were also enriched in pathways related to environmental adaptation, organismal systems and DNA repair and recombination (**Supplementary Figure 4B**). The prediction of miRNA–NLR interactions identified 175 of the178 NLR genes as targets of 311 distinct miRNA families at an expectation value threshold of < 3, involving both cleavage and translational repression (**Figure 2D**). Among these, the miR482 family exhibited the highest targeting capacity, with 113 target sites across 106 NLR genes, followed by miR1507 family with 34 target sites. At the gene level, *Vacg2168* was the most extensively targeted, with 13 target sites recognized by 12 different miRNAs. *Vacg12000*, *Vacg13418*, *Vacg13543*, and *Vacg18379*, each contained 10 target sites (**Figure 2E**; **Supplementary Table 11****).**

**Figure 2:**
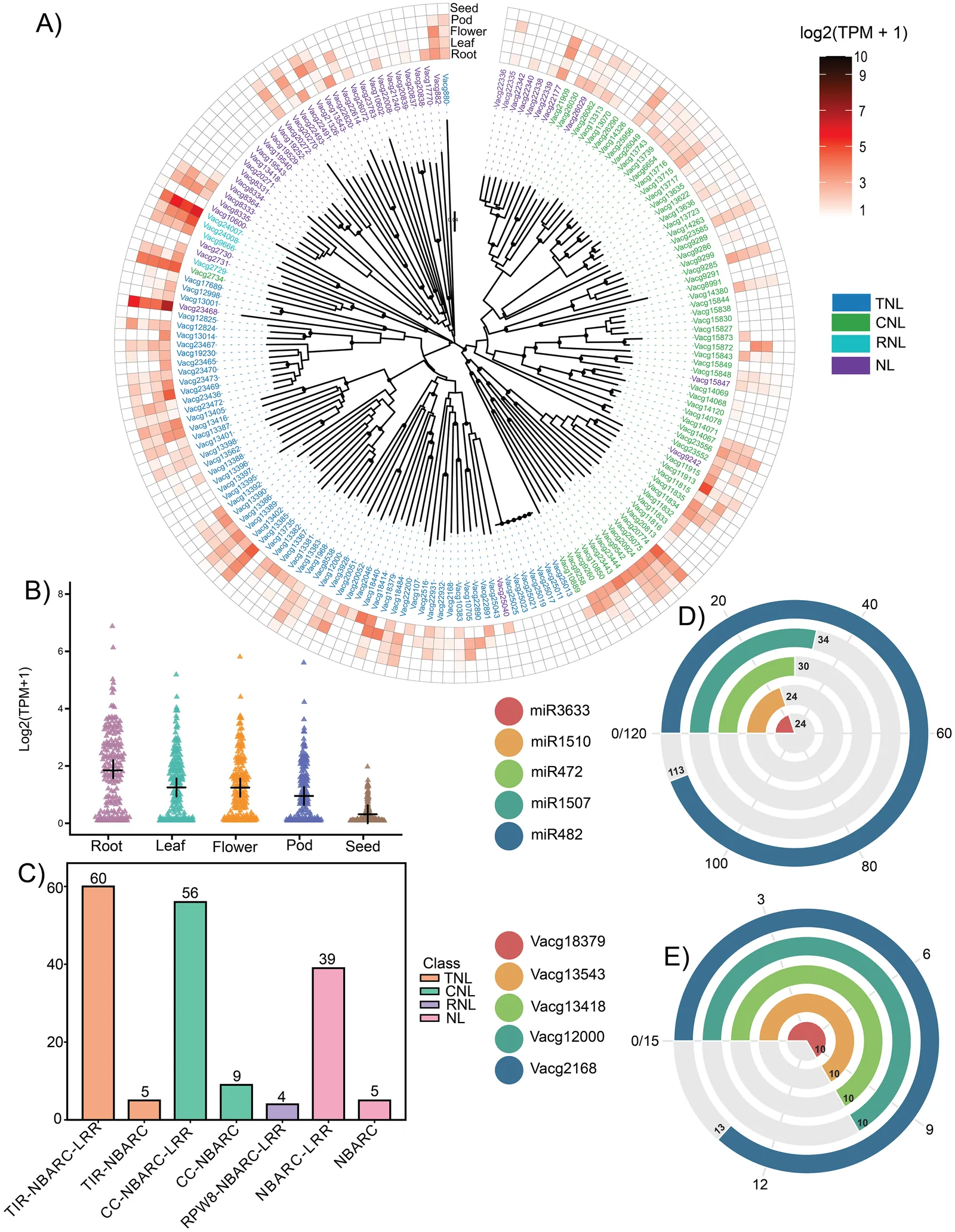
Phylogenetic and transcriptomic analyses of NLR genes in *Vigna aconitifolia* cv TMV (Mb) 1. **(A)** Neighbor-joining phylogenetic tree of moth bean NLR proteins. The heatmap adjacent to the tree indicates relative gene expression levels across root, leaf, flower, pod, and seed. Gene IDs are color-coded to represent TNL, CNL, RNL, and NL subfamilies, **(B)** Jitter plot showing transcript abundance (TPM) of NLR genes across five tissues: root, leaf, flower, pod, and seed. Statistical significance among tissues was determined using one-way ANOVA followed by Tukey’s HSD test, **(C)** Bar plot showing the number of genes in different type of NLR classes, **(D)** Circular bar plot showing the top miRNA families targeting NLR genes, and **(E)** Circular bar plot showing the top NLR genes targeted by miRNA families.

### 3.5 Expression pattern of flavonoid biosynthesis genes

A total of 54 genes encoding key enzymes of the flavonoid biosynthesis pathway were identified, and their expression patterns were analysed across five tissues (root, leaf, flower, pod, and seed) using RNA-seq data (**Figure 3**; **Supplementary Table 12**). Flavonoid biosynthesis begins from the phenylpropanoid pathway. Genes involved in the upstream phenylpropanoid pathway, including phenylalanine ammonia-lyase (PAL), cinnamate 4-hydroxylase (C4H), and 4-coumarate-CoA ligase (4CL) showed predominantly high expression in roots. Among these, *Vacg6571* (PAL), *Vacg19530* (C4H) and *Vacg3764* (4CL) exhibited the highest transcript abundance in root tissue. The major flavonoid biosynthesis enzyme chalcone synthase (CHS), showed tissue-specific expression, with *Vacg16162* highly expressed in leaves and pods, whereas *Vacg9015* showed predominant expression in roots. The isoflavonoid pathway, a specialized branch of flavonoid biosynthesis predominantly found in legumes. Among the pathway genes, isoflavone synthase (IFS) gene *Vacg1704* exhibited the highest transcript abundance in roots, indicates active transcriptional activity for isoflavonoid biosynthesis genes in root. Similarly, chalcone reductase (CHR) genes *Vacg20788* and *Vacg20299*, together with the chalcone isomerase (CHI) genes *Vacg4526, Vacg4525*, and *Vacg6866*, were also highly expressed in root tissue, indicating coordinated expression of the isoflavonoid pathway. Within the downstream flavonoid pathway, flavone synthase II (FNSII) gene *Vacg12384* showed relatively higher expression in flowers, whereas the flavanone 3-hydroxylase (F3H) (*Vacg2589*) and flavonol synthase (FLS) (*Vacg24566*) genes were mainly expressed in leaves. Additionally, dihydroflavonol 4-reductase (DFR, *Vacg7916*), anthocyanidin synthase (ANS, *Vacg9975*), isoflavone 2’-hydroxylase (I2’H, *Vacg1470*), and chalcone reductase (CHR, *Vacg14079*) genes also showed distinct tissue-specific expression patterns. To validate the RNA-seq results, seven genes (*Vacg1470, Vacg1704, Vacg2589*, *Vacg7916, Vacg9975, Vacg14079*, and *Vacg4526*) were selected from the genes described above and analysed by qRT-PCR. Their expression profiles were consistent with the RNA-seq data, confirming the reliability of the transcriptome analysis and supporting the observed tissue-specific expression profiles of flavonoid biosynthesis genes.

**Figure 3:**
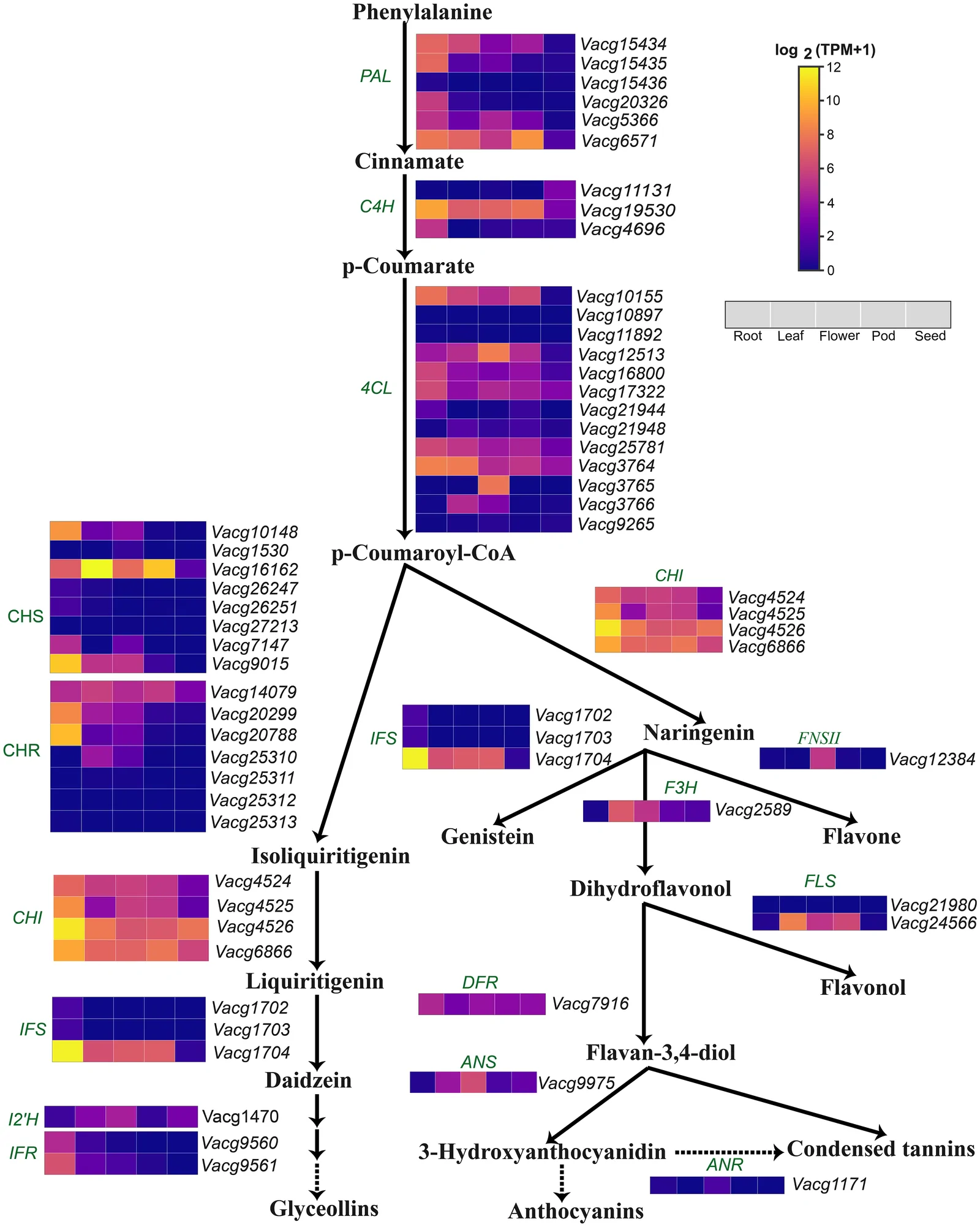
Expression profiles of genes involved in flavonoid and isoflavonoid biosynthesis in moth bean. Heatmaps show the relative expression levels of candidate genes across five tissues (root, leaf, flower, pod, and seed). Gene expression levels were calculated as transcripts per million (TPM) and visualized as log_2_ (TPM + 1). Dark purple and yellow colors indicate low and high expression levels, respectively. Abbreviations: PAL, phenylalanine ammonia-lyase; C4H, cinnamate 4-hydroxylase; 4CL, 4-coumarate-CoA ligase; CHS, chalcone synthase; CHI, chalcone isomerase; CHR, chalcone reductase; IFS, isoflavone synthase; F3H, flavanone 3-hydroxylase; FNSII, flavone synthase II; FLS, flavonol synthase; DFR, dihydroflavonol 4-reductase; ANS, anthocyanidin synthase; ANR, anthocyanidin reductase; I2’H, isoflavone 2′-hydroxylase; IFR, isoflavone reductase.

## 4 Discussion

In the present study, we report the highly contiguous genome assembly of moth bean. The estimated genome size of TMV (Mb) 1 (448.98 Mb) is slightly smaller than the previously reported genome size of ~469 Mb for the moth bean accession PI426980 (Diakostefani et al., 2024). The smaller genome size likely results from genotype variation or methodological differences. The observed genome size variation seems to represent an inherent evolutionary pattern within *Vigna*, may driven by lineage-specific differences in repetitive element content, structural rearrangements, and historical genome duplication events. In our study, the TMV (Mb) 1 genome was assembled to a total size of ~420.3 Mb, representing 93.63% of the estimated genome size, with a contig N50 of 22.3 Mb and maximum contig length of 59.6 Mb. The contig N50 obtained in our study is higher than the values reported for *V. radiata* (~10.35 Mb) (Oraron et al., 2025), *V. mungo* (~5.20 Mb) (Pootakham et al., 2021), *V. angularis* (16.50 Mb) (Li et al., 2024) and *V. umbellata* (~18.26 Mb) (Guan et al., 2022). N50 indicates assembly continuity and fragmentation, with higher values indicating improved assembly quality. However, several of these genomes have been refined to chromosome scale through Hi-C–based scaffolding, limiting direct comparisons of contiguity metrics. Future chromosome-scale assembly will complement the current high-quality contig assembly and facilitate more comprehensive analyses of genome structure and evolution. BUSCO analysis of the TMV (Mb) 1 genome recovered >98% of conserved single-copy orthologues, supports assembly completeness. Assembly integrity was further supported by read mapping, with Illumina genomic reads aligning at 99.63% and tissue-derived mRNA reads at 97.04%. These high mapping rates collectively confirm the accuracy, completeness and reliability of the TMV (Mb) 1 genome assembly. Comparative genomic analysis revealed strong conservation of the moth bean genome, with 98.2% of genes assigned to shared families across related species. The identification of a small number of moth bean–specific gene families points to recent lineage-specific innovations that may contribute to species-adapted traits. Phylogenetic analysis based on single-copy orthologs placed moth bean as sister to the *V.radiata*–*V. mungo* clade, diverging ~3.7 Mya (95% HPD: 2.7–5.1 Mya). This evolutionary basis supports translational genomics and trait-focused crop improvement. The expansion of 776 and contraction of 383 gene families indicate lineage-specific evolutionary changes in moth bean. Expanded families were enriched in ribosome biogenesis, RNA processing, translation, proteasome activity and circadian rhythm, suggesting enhanced regulation of protein homeostasis and environmental responses. Conversely, contracted families associated with membrane transport, defense and specialized metabolism may reflect evolutionary reorganization of these functions. Together, these patterns suggest gene-family turnover contributed to moth bean diversification, although its role in stress adaptation requires further functional validation.

Repetitive elements contribute to genome size variation, structural organization and gene regulation in plants. They also shape chromatin architecture, recombination patterns and adaptive evolution across plant species (Mehrotra and Goyal, 2014). Repetitive elements account for 212.97 Mb (50.66%) of the TMV (Mb) 1 genome, a repeat content comparable to that of *V. unguiculata* (49.5%) (Lonardi et al., 2019). This repeat content is lower than those reported for *V. umbellata* (57.19%) (Guan et al., 2022), *V. angularis* (54.50% - 243.62 Mb) (Li et al., 2024), *V. radiata* (54.76% - 253.85 Mb) (Khanbo et al., 2024), but higher than those of *V. reflexo-pilosa* (44.44% -438.37 Mb) (Pootakham et al., 2023), and *V. mungo* (41.1%-205.4 Mb) (Pootakham et al., 2021). In total, 27,434 protein-coding genes were predicted, a number comparable to that reported for closely related species *V. radiata* (Jia et al., 2025) and *V. mungo* (Pootakham et al., 2021). These genes were distributed across 34 contigs, with an average density of 806.88 genes per contig. The gene models showed high BUSCO completeness (99%), indicating near-complete conserved gene representation. Functional annotation assigned roles to 27,411 (99.91%) of the 27,434 predicted protein-coding genes (99.91%), providing a valuable resource for molecular breeding and functional genomics of stress tolerance and nutritional traits in moth bean. We also identified 1,999 genes encoding putative transcription factors in the moth bean genome, classified into 58 families. Transcription factors play central roles in regulating defense responses and secondary metabolite biosynthesis (Patra et al., 2013; Viswanath et al., 2023; Liu et al., 2024a). The bHLH family (180 genes) was the most abundant, followed by MYB (179) and ERF (152). bHLH transcription factors are widely associated with plant growth, development and stress responses (Qian et al., 2021; Zuo et al., 2023; Gao and Dubos, 2024), and have key roles in secondary metabolite biosynthesis (Gao et al., 2022; Wang et al., 2025). Furthermore, a total of 7,752 non-coding RNAs (ncRNAs), including 126 miRNAs, were identified. ncRNAs regulate gene expression at transcriptional and post-transcriptional levels (Chao et al., 2022). miRNAs, in particular, control plant growth, development and stress responses (Dong et al., 2022; Samynathan et al., 2023). Their identification in moth bean provides a resource for studying stress adaptation and supporting breeding efforts.

Resistance to pathogen infection in plants is mediated by disease-resistance (R) genes, which encode proteins that recognize invading pathogens (Ding et al., 2022; Tailor and Bhatla, 2024; Du et al., 2025). Many validated R genes belong to the NLR family, classified into subgroups based on variation in domain architecture (McHale et al., 2006). A total of 178 NLR genes were identified in the moth bean genome, many organized in genomic clusters that may enable coordinated regulation, rapid pathogen recognition and activation of downstream defense responses to pathogen attacks. These genes showed differential abundance across tissues, suggesting roles in defense responses in moth bean. Although NLR resistance genes in *Vigna* species remain poorly characterized, comparative analysis with *Arabidopsis thaliana* identified homologs of resistance genes involved in pathogen defense responses. For instance, *AT5G36930*, which encodes a TIR-NBS-LRR disease resistance protein, was homologous to *Vacg13367*, *Vacg13381*, and *Vacg13382*, whereas *AT3G14470*, which encodes an NB-ARC domain-containing disease resistance protein, was homologous to *Vacg22177*, *Vacg22335*, *Vacg22336*, *Vacg22338*, and *Vacg2233*. These homologs are promising candidates for functional validation and potential targets for breeding enhanced disease resistance. Furthermore, miRNA–NLR interaction analysis revealed that 175 genes were targeted by 311 miRNA families, miR482 family showed the highest targeting frequency, followed by miR1507. miR482 and miR1507 constitute a distinct miRNA superfamily in plants, with precursor sequences showing significant similarity to NLR genes. These miRNAs target NLR transcripts and modulate plant immune responses (Zhai et al., 2011; Liao et al., 2022). miR482 functions as a key post-transcriptional regulator of plant immunity by targeting conserved sequences encoding the P-loop motif of NBS–LRR proteins (Li et al., 2012; Zhang et al., 2016). In *V. mungo*, an NLR-encoding resistance gene targeted by miR482 has been reported and, based on circumstantial evidence, was proposed to encode a protein that recognizes a viral effector (Kundu et al., 2017). miR1507 similarly contributes to the regulation of plant defense responses by triggering the production of phased secondary small interfering RNAs (phasiRNAs). In soybean, miR1507-derived phasiRNAs regulate the expression of defense-associated NLR genes during *Phytophthora sojae* infection (Wong et al., 2014). At the individual gene level, *Vacg2168* was the most extensively regulated, being targeted by 12 miRNAs with 13 predicted target sites. Genes *Vac*g12000, *Vac*g13418, *Vac*g13543, and *Vac*g18379 followed, each containing 10 predicted target sites. These findings highlight post-transcriptional regulation of NLR genes, providing insights into plant immunity and supporting resistance breeding strategies.

Despite the important roles of flavonoids in plant adaptation and nutritional quality, genes underlying flavonoid biosynthesis in moth bean remain poorly characterized. In plants, flavonoid biosynthesis originates from the phenylpropanoid pathway, which provides precursors for secondary metabolites involved in stress tolerance and plant–microbe interactions (Ferrer et al., 2008; Vogt, 2010). In this study, genes encoding phenylpropanoid pathway enzymes, including *PAL* (*Vacg6571*), *C4H* (*Vacg19530*), and *4CL* (*Vacg3764*), showed high expression in root tissues, indicating active precursor formation. Similarly, flavonoid and isoflavonoid pathways enzymes, such as *CHS* (*Vacg9015*), *CHR* (*Vacg20788* and *Vacg20299*), and *CHI* (*Vacg4526*, *Vacg4525*, and *Vacg6866*), were also highly expressed in roots compared with other tissues. This pattern suggests that roots are a major site of flavonoid biosynthesis in moth bean, consistent with reports in other legumes (Liu et al., 2024b; Wang et al., 2024). However, elevated transcript abundance does not necessarily correspond directly to increased enzyme activity or metabolite accumulation, as flavonoid biosynthesis may also be influenced by post-transcriptional, translational, and post-translational regulatory mechanisms. Therefore, direct metabolite profiling and/or enzyme activity measurements would be required to confirm whether the observed transcriptional activity results in higher flavonoid accumulation in roots. In legumes, flavonoids function as signaling molecules in rhizosphere communication and defense responses (Qiao et al., 2024; Kumar et al., 2024; Lin et al., 2024), supporting roles in stress tolerance and environmental adaptation. *FNSII* (*Vacg12384*) showed relatively higher expression in flowers, whereas *F3H* (*Vacg2589*) and *FLS* (*Vacg24566*) were predominantly expressed in leaves, indicating active flavonol biosynthesis in photosynthetic tissues. Flavonols in leaves act as UV-protectants and antioxidants, protecting cells from photooxidative damage. Elevated expression of these genes is associated with enhanced flavonoid production under environmental stresses such as intense light and UV-B radiation (Liu et al., 2026; Parveen et al., 2025). Additionally, anthocyanin biosynthesis, including ANS (*Vacg9975*) and DFR (*Vacg7916*), showed moderate expression in flowers, suggesting roles in pigmentation (Pal et al., 2023; Gao et al., 2024). Furthermore, identified transcription factors, such as the MYB and bHLH families, may regulate flavonoid biosynthesis, as they are established regulators of phenylpropanoid and flavonoid pathways in plants (Xu et al., 2015). Several genes encoding MYB (*Vacg2808*, *Vacg9732*, & *Vacg16323*) and bHLH (*Vacg3281*, *Vacg17512*, & *Vacg23638*) transcription factors exhibited distinct tissue-specific expression patterns, particularly those corresponding with flavonoid structural genes, highlight them as promising candidate regulators. However, these regulatory relationships remain putative and require further studies. Collectively, our study findings provide insights into flavonoid biosynthesis and support future functional and breeding studies in moth bean.

In summary, this study provides the first highly contiguous genome assembly of moth bean, establishing a genomic framework for the annotation of disease-resistance genes and the investigation of flavonoid biosynthesis. Although not yet resolved at chromosome scale, this resource provides a foundation for genome-wide analyses, functional genomics studies, and molecular breeding in this underutilized legume. Future chromosome-scale assembly through Hi-C sequencing or optical mapping will enable comprehensive analyses of genome organization, gene clustering, structural variation, and synteny with other *Vigna* species, further increasing the utility of this resource for genetic improvement.

## Data Availability

The data that support the findings of this study are available from the corresponding author (AK) upon request. All sequencing data have been deposited in the NCBI Sequence Read Archive (SRA) under BioProject accession PRJNA1420186. The corresponding SRA accession numbers are SRR37153845- SRR37153851.

## Supplementary material

Supplementary Figure 1 Genome assembly and protein set completeness were assessed using BUSCO with Embryophyta, Eudicots, and Fabales lineage datasets.

Supplementary Figure 2 Gene ontology enrichment analysis of expanded **(A)** and contracted **(B)** gene families in moth bean.

Supplementary Figure 3 KEGG pathway enrichment analysis of expanded **(A)** and contracted **(B)** gene families in moth bean.

Supplementary Figure 4 Gene ontology **(A)** and KEGG enrichment **(B)** analyses of NLR genes in moth bean.
