# Supplementary material for: High-contiguity genome assembly of moth bean (Vigna aconitifolia) reveals genes for disease resistance and flavonoid biosynthesis
Source: Front Plant Sci. 2026 Sep 21;17:1932838. doi: 10.3389/fpls.2026.1932838 (PMC13635161; doi:10.3389/fpls.2026.1932838)
Supplement: Supplementary file 1 [file Table1.docx]

**Supplementary Table 1.** The primers used for qRT-PCR analysis

| **S.No** | **Gene ID** | **Forward sequence 5’-3’** | **Reverse sequence 5’-3’** | **Reference** |
| --- | --- | --- | --- | --- |
| 1 | Vacg1470 | GGCAACCTCCACCTTATCAA | GAGGTGATGACGACCACAAA | This study |
| 2 | Vacg1704 | AGTGTGAAATTGACGGGTATGT | CCAATGGTCTGTCCCAGTATTT | This study |
| 3 | Vacg2589 | GACTGAGGAATACAGCGAGAAG | CACAACCTTCTGGTCCATATCA | This study |
| 4 | Vacg7916 | TCAAAGGTCGCTGCTGATAAA | CTTGCCAGGCCCATAGATTAC | This study |
| 5 | Vacg9975 | CTGAGGCTACAAGCGAGTATG | CCACCCACTTCCTTCTCTAATC | This study |
| 6 | Vacg14079 | TCATCTTGTTCTTCCTGCTCTG | CTCCAGGTTTCACACTGATAGG | This study |
| 7 | Vacg4526 | GGGACATATGGTGATGCTGAA | CGGGTGATTGCCTGTAGAAA | This study |
| 8 | VaActin | CTGCTGGCATTCATGAGACC | ACCACCACTGAGAACGATGT | Suranjika et al. 2022 |

**Supplementary Table 2.** Summary of DNA sequencing and RNA sequencing data

| **Tissue** | **Library**  **type** | **Library size** | **Number of**  **reads** | **Number of bases (Gb)** | **Average Read length (bp)** | **Platform** | **Sequence**  **coverage^*^** | **Accession**  **number** |
| --- | --- | --- | --- | --- | --- | --- | --- | --- |
| **DNA sequences** | | | | | | | | |
| Leaf | Single end | 18-20 kb | 2026331 | 37.04 | 18280 | PacBio- Revio | 82.50X |  |
| Leaf | Paired end | 350 bp | 208561337 | 31.28 | 150 | Iluumina nova seq 6000 | 69.67X |  |
|  |  | **RNA sequences** | | | | | | |
| Root | Paired end | 150 bp | 43,632,102 | 6.94 | 150 | Iluumina nova seq 6000 | - |  |
| Leaf | Paired end | 150 bp | 42,858,408 | 6.81 | 150 | Iluumina nova seq 6000 | - |  |
| Flower | Paired end | 150 bp | 43,824,849 | 6.97 | 150 | Iluumina nova seq 6000 | - |  |
| Pod | Paired end | 150 bp | 44,454,161 | 7.07 | 150 | Iluumina nova seq 6000 | - |  |
| Seed | Paired end | 150 bp | 42,464,324 | 6.75 | 150 | Iluumina nova seq 6000 | - |  |
|  |  |  | **Total** | 34.54 |  |  |  |  |

^*^The estimated genome size was ~448.98 Mb

**Supplementary Table 3.** Statistics of the 21-mer analysis of the moth bean cv TMV (Mb) 1 genome

| **Kmer**  **length** | **Kmer**  **numbers** | **Kmer**  **depth** | **Genome size**  **(bp)** | **Heterozygous**  **(%)** | **Repeat**  **(%)** |
| --- | --- | --- | --- | --- | --- |
| 21 | 1256517795 | 74 | 448.98 | 0.138 | 43.10 |

**Supplementary table 4.** Evaluation of genome coverage using Illumina DNA short reads and RNA sequencing data from different tissues of
moth bean TMV (Mb) 1

| **Tissue** | **Number of reads** | **Clean reads** | **Mapped** **reads** | **Mapping rate**  **(%)** | **Average depth**  **(×)** | **Genome**  **coverage at 10× (%)** |
| --- | --- | --- | --- | --- | --- | --- |
| **DNA sequences** | | | | | | |
| Leaf | 208561337 | 206679763 | 207785530 | 99.63 | 73.55 | 97.13 |
| **RNA sequences** | | | | | | |
| Root | 43,632,102 | 40,278,792 | 38068073 | 94.51 | - | - |
| Leaf | 42,858,408 | 40,141,467 | 37324941 | 92.98 | - | - |
| Flower | 43,824,849 | 41,683,849 | 41285597 | 99.04 | - | - |
| Pod | 44,454,161 | 42,365,597 | 42093208 | 99.36 | - | - |
| Seed | 42,464,324 | 40,054,454 | 39792211 | 99.35 | - | - |
|  |  |  | **Average** | 97.04 |  |  |

**Supplementary table 5.** Classification of transcription factors in the TMV (Mb) 1 genome assembly.

| **Transcription factor family** | **Gene number** | **Transcription factor family** | **Gene number** | **Transcription factor family** | **Gene number** |
| --- | --- | --- | --- | --- | --- |
| AP2 | 24 | GRF | 9 | Nin-like | 13 |
| ARF | 28 | GeBP | 7 | RAV | 3 |
| ARR-B | 18 | HB-PHD | 3 | S1Fa-like | 3 |
| B3 | 41 | HB-other | 8 | SAP | 1 |
| BBR-BPC | 5 | HD-ZIP | 55 | SBP | 23 |
| BES1 | 7 | HRT-like | 1 | SRS | 11 |
| C2H2 | 121 | HSF | 33 | STAT | 2 |
| C3H | 41 | LBD | 48 | TALE | 31 |
| CAMTA | 8 | LFY | 1 | TCP | 27 |
| CO-like | 13 | LSD | 4 | Trihelix | 41 |
| CPP | 6 | M-type_MADS | 29 | VOZ | 3 |
| DBB | 11 | MIKC_MADS | 45 | WOX | 121 |
| Dof | 41 | MYB | 179 | WRKY | 94 |
| E2F/DP | 7 | MYB_related | 69 | Whirly | 3 |
| EIL | 5 | NAC | 91 | YABBY | 9 |
| ERF | 152 | NF-X1 | 2 | ZF-HD | 18 |
| FAR1 | 36 | NF-YA | 9 | bHLH | 180 |
| G2-like | 52 | NF-YB | 20 | bZIP | 85 |
| GATA | 29 | NF-YC | 15 |  |  |
| GRAS | 56 | NZZ/SPL | 2 |  |  |

**Supplementary table 6.** Summary of genes and orthogroups for species used in this study

| **S. No** | **Species** | **Number**  **of genes** | **Number of orthogroups** | **Number of genes**  **in orthogroups** | **Number of species specific orthogroups** | **Number of genes in species specific orthogroups** | **Single copy**  **genes** |
| --- | --- | --- | --- | --- | --- | --- | --- |
| 1 | *Arabidopsis thaliana* | 27154 | 13350 | 24650 | 591 | 2713 | 8175 |
| 2 | *Cajanus cajan* | 27142 | 14545 | 26640 | 64 | 151 | 8804 |
| 3 | *Cicer arietinum* | 23674 | 14183 | 23399 | 46 | 141 | 9477 |
| 4 | *Oryza sativa* | 34865 | 13774 | 27437 | 1454 | 5901 | 8079 |
| 5 | *Pisum sativum* | 35055 | 14495 | 34212 | 193 | 1923 | 9201 |
| 6 | *Vigna angularis* | 25527 | 14390 | 25352 | 21 | 49 | 9097 |
| 7 | *Vigna umbellata* | 24089 | 14025 | 23732 | 16 | 38 | 8721 |
| 8 | *Vigna unguiculata* | 26639 | 14446 | 26358 | 29 | 70 | 8991 |
| 9 | *Vigna stipulacea* | 23802 | 14208 | 23676 | 11 | 26 | 8946 |
| 10 | *Vigna trilobata* | 24920 | 14442 | 24118 | 36 | 83 | 9131 |
| 11 | *Vicia faba* | 31793 | 14691 | 29525 | 409 | 1298 | 8597 |
| 12 | *Vigna radiata* | 29961 | 15035 | 29061 | 97 | 251 | 8263 |
| 13 | *Lotus japonicus* | 31588 | 14473 | 30916 | 229 | 1202 | 8494 |
| 14 | *Vigna aconitifolia* | 27434 | 15118 | 26934 | 27 | 85 | 9161 |
| 15 | *Vigna mungo* | 29147 | 15261 | 28339 | 86 | 209 | 9242 |
